# Microarray analysis of infectious bronchitis virus infection of chicken primary dendritic cells

**DOI:** 10.1186/s12864-019-5940-6

**Published:** 2019-07-08

**Authors:** Jian Lin, Zhisheng Wang, Jialu Wang, Qian Yang

**Affiliations:** 1 College of Life Sciences, Nanjing Agricultural University, Wei gang 1, Nanjing, Jiangsu 210095 People’s Republic of China; 2 College of Veterinary medicine, Nanjing Agricultural University, Wei gang 1, Nanjing, Jiangsu 210095 People’s Republic of China; 3 National Veterinary Product Engineering Research Center, Jiangsu Academy of Agricultural Sciences, Nanjing, China

**Keywords:** Infectious bronchitis virus, Dendritic cells, mRNA, microRNA, lncRNA

## Abstract

**Background:**

Avian infectious bronchitis virus (IBV) is a major respiratory disease-causing agent in birds that leads to significant losses. Dendritic cells (DCs) are specialised cells responsible for sampling antigens and presenting them to T cells, which also play an essential role in recognising and neutralising viruses. Recent studies have suggested that non-coding RNAs may regulate the functional program of DCs. Expression of host non-coding RNAs changes markedly during infectious bronchitis virus infection, but their role in regulating host immune function has not been explored. Here, microarrays of mRNAs, miRNAs, and lncRNAs were globally performed to analyse how avian DCs respond to IBV.

**Results:**

First, we found that IBV stimulation did not enhance the maturation ability of avian DCs. Interestingly, inactivated IBV was better able than IBV to induce DC maturation and activate lymphocytes. We identified 1093 up-regulated and 845 down-regulated mRNAs in IBV-infected avian DCs. Gene Ontology analysis suggested that cellular macromolecule and protein location (GO-BP) and transcription factor binding (GO-MF) were abundant in IBV-stimulated avian DCs. Meanwhile, pathway analysis indicated that the oxidative phosphorylation and leukocyte transendothelial migration signalling pathways might be activated in the IBV group. Moreover, alteration of microRNAs (miRNAs) and long non-coding RNAs (lncRNAs) was detected in IBV-stimulated avian DCs. In total, 19 significantly altered (7 up and 12 down) miRNAs and 101 (75 up and 26 down) lncRNAs were identified in the IBV-treated group. Further analysis showed that the actin cytoskeleton and MAPK signal pathway were related to the target genes of IBV-stimulated miRNAs. Finally, our study identified 2 TF-microRNA and 53 TF–microRNA–mRNA interactions involving 1 TF, 2 miRNAs, and 53 mRNAs in IBV-stimulated avian DCs.

**Conclusions:**

Our research suggests a new mechanism to explain why IBV actively blocks innate responses needed for inducing immune gene expression and also provides insight into the pathogenic mechanisms of avian IBV.

**Electronic supplementary material:**

The online version of this article (10.1186/s12864-019-5940-6) contains supplementary material, which is available to authorized users.

## Background

Avian infectious bronchitis virus (IBV) is a member of the coronaviridiae family that targets both the meat-type and commercial egg-laying chickens. IBV mainly replicates in the epithelial cells of the respiratory tract, causing lower respiratory tract infections in chickens [1, 2]. The threat of IBV infection is global, as this respiratory virus can cause acute and contagious respiratory infections in poultry, resulting in serious economic losses [3, 4]. The strategy of reducing IBV infection through vaccination has been recognised as a viable option to control IBV and has been somewhat successful [5, 6]. The continuing prevalence of IBV might be attributable to divergent IBV subtypes. Despite efficient protection against genetically similar strains, vaccines may not be effective against all subtypes [7, 8]. Isolated IBV has been associated with proventriculus and kidney lesions [9]. IBV infections are difficult to control mainly due to poor cross-protection between different types of IBV [10, 11]. Thus, effective control of IBV infection is still a major challenge for poultry health management.

IBV has a large genome and is an obligate intracellular pathogen that exploits the host’s cellular machinery to replicate [12, 13]. Elucidating the potential cellular interactions between the host immune system and the pathogen will not only provide insights into disease pathogenesis but will also support strategies for preventing IBV infection [10, 14, 15]. Previous studies of IBV have focussed primarily on Vero cells and avian DF-1 cells [16, 17] and have rarely involved the function of avian DCs during IBV infection. Avian DCs have the unique ability to induce both innate and acquired immune responses and are mainly identified in the primary and secondary lymphoid organs [18, 19]. Our previous study illustrated that the TF-microRNA-mRNA regulation loop might mediate the activation of avian DCs stimulated by infectious bursal disease virus (IBDV) [20].

Non-coding RNAs are post-transcriptional regulators that fine-tune the immune response [21, 22]. Among non-coding RNAs, miRNAs are key regulators of immune responses [23, 24]. Unsurprisingly, major changes in miRNA expression have been shown to be the underlying mechanism triggering the host immune response [24]. For example, miR29c was found to enhance the immune function of DCs, whereas miR375 was suggested to attenuate the antigen-presenting ability of mouse DCs [25, 26]. In addition to evoking an immune response, miRNAs may also shape host–virus interactions and defend against viral infection [27, 28]. For example, miR674 can target host Mbnl3 to modulate antiviral responses inhibiting H9N2 avian influenza virus replication [29]. Meanwhile, lncRNAs play essential roles in regulating the defence process against viral infection [21]. To explore the potential roles of miRNAs and lncRNAs in IBV infection, we first evaluated the ability of IBV to induce chicken and mouse DC maturation and lymphocyte cell activation. Then, microarrays and bioinformatic analysis were performed to reveal the mechanism underlying the IBV response in avian DCs. Our results provide a better mechanism of the interaction between IBV and avian dendritic cells.

## Methods

### Animal and virus

Our mice, including all the 6 to 8 wk. SPF BALB/c or C57BL/6, were purchased from Yang Zhou University (Center Comparative Medical). Whilst 3 to 4 wk. SPF ROSS 308 avian were bought from the Jiangsu Academy of Agricultural Sciences (JAAS) (Nanjing, China). All animals were maintained at an animal facility under pathogen free conditions. IBV strain Massachusetts-41 [30] containing 1 × 10^6.6^ EID_50_ (egg infectious dose) of IBV was kindly provided by Nanjing Tian bang Bio-Industry Co. Ltd. (Nanjing, China). The infectious bronchitis virus was inactive by UV light (260 nm, with in 15 cm) for 4 h and tested for complete loss of the infectivity before using.

### Phenotypic alteration and T-cell proliferation of DCs stimulated by IBV

#### Surface marker analysis of avian bone marrow-derived dendritic cells (BMDCs)

Avian bone marrow-derived dendritic cells were obtained from 3 to 4 week-old chicks. All animal experiments were carried out in accordance with the regulations and guidelines of laboratory animals of Nanjing Agriculture University (Nanjing, China). Initially, avians were euthanized by putting the cultured cage of avian into the carbon dioxide sealed tank. Then, femurs and tibias were removed and isolated from the surrounding muscle tissue using sterile instruments (we did not using any anesthesia during the removal of avian femurs and tibias from animals for the avian for the animal has been euthanized). The detail method for isolating avian BMDCs were the same as our previous published manusccript [31]. Immature avian BMDCs were plated in fresh medium (1 × 10^6^ cells/ml) and treated with LPS (1 μg/ml, positive control), IBV (EID_50_ = 10^–6.5^/0.1 ml) and inactivated IBV for 24 h at 37 °C and 5% CO_2_. For IBV, cells were incubated at a multiplicity of infection of 1 (MOI = 1) for 24 h. Then, both group were collected, washed and incubated at 4 °C for 30 min with PE-conjugated anti-human CD11c, anti-chicken CD40 (clone: AV79) or CD86 (clone: IAH:F853:AG2) antibody. Cells stained by CD40 and CD86 were then stained by PE-conjugated goat anti-mouse IgG secondary antibody (MultiScience, China) diluted 1: 5000 for another 15 min at room temperature. Cells were washed and analyzed with Fluorescence Activated Cell Sorter (FACS).

#### Surface marker analysis of mice BMDCs

Immature mice BMDCs were plated in fresh medium (1 × 10^6^ cells/ml) and treated with LPS, IBV or inactivated IBV for 24 h at 37 °C and 5% CO_2_. Cells were collected, washed and incubated at 4 °C for 30 min with following antibody (monoclonal antibodies (anti-mouse CD11c (N418), anti-mouse CD40(1C10), anti-mouse CD86(GL1), anti-mouse MHCII (M5/114.15.2) and anti-mouse CD80 antibody (16-10A1), respectively (eBioscience, USA)), and analyzed by FACS.

#### T-cell proliferation assays

To further evaluate the antigen presenting function of avian or mouse DCs, mixed lymphocyte reaction (MLR) experiments were performed as previous research. The detail method for isolating avian BMDCs were the same as our previous published manusccript [32]. The stimulator cells were DCs previously treated with IBV, inactivated IBV or LPS for 24 h. All experiments were performed at least in triplicates. After 3 days of culture in 5% CO_2_ at 37 °C, cell proliferation was determined by Cell Counting Kit-8 (CCK-8) assay (Beyotime, China). Each well received 20 μL CCK-8 solution and was incubated for 2 h at 37 °C before absorbance measurement at 450 nm. The Stimulation Index was calculated as references.

### Microarrays analyses of mRNAs, microRNAs and lncRNAs

Cultured avian BMDCs were randomly divided into either control or IBV-stimulated groups. For IBV stimulated group, cells were incubated at a multiplicity of infection of 1 (MOI = 1) for 12 h. Each group consisted of three wells of BMDCs from three chickens. Total RNA and microRNA were separately isolated using the RNeasy Total RNA Isolation Kit (QIAGEN, Germany). The avian microarray (containing mRNA and lncRNA, RiboArray™ Custom Array (A10000–1-90)) was hybridised. Approximately 3900 lncRNAs and 15,081 mRNAs are detected using the mRNA microarray. In each microarray, we have three replication per slide. Also, we have used the microRNA array (A10000–1-40) to detect the experssion of microRNA. Approximately 991 microRNAs are detected using the microRNA microarray Raw data were normalized using the RMA method [20, 33].

#### Identification and bioinformatics analyses of differentially expressed mRNAs

Differentially expressed (DE) mRNAs between control avian DCs group and IBV stimulated group were determined with a cut off of at least 2-fold change and a *P* value less than 0.01. Such genes were subject to GO categorization, KEGG (Kyoto Encyclopedia of Genes and Genomes) and BioCartapathway analyses. Analyses were performed with DAVID (the Database for Annotation, Visualization and Integrated Discovery) by using an independent list of differentially-expressed genes.

#### Identification of differentially expressed microRNAs and its target prediction and bioinformatics analyses

DE microRNAs were chosen with a cut off of at least 2-fold change and a *p* value less than 0.05. Potential targets of these microRNAs were predicted using the microRNA target prediction and functional study database (miRDB) and TargetScan. Taking the intersection of these two predictions, we obtain the optimal potential target genes. To further understand the potential functions of microRNA - target genes, GO categorization and pathway analysis were assigned using the DAVID gene annotation tool.

#### Identification of differentially expressed lncRNAs and its association analyses with differentially expressed mRNAs

DE lncRNAs between IBV stimulated group and control group were first chosen with a cut off of at least 2-fold change and a *P* value less than 0.01. Since transcriptional regulation by lncRNAs could work either in cis or trans model, we then predicted the cis and trans target gene of difference expressed lncRNAs as previous published manuscript [34]. To further classifly lncRNA trans-target genes, the RNAplex program (RNAplex < − 100) was then used to identify possible trans-target genes of the lncRNAs. Thirdly, the Pearson correlation coefficient absolute value over 0.9 together with P value less than 0.01 were used to predict the lncRNA’s co-expression target genes. Finally, the lncRNAs and co-expression genes relationship networks were drawn using Cytoscape software [35].

### QRT-PCR confirmation of mRNAs, microRNAs and lncRNAs microarrays result

Based on our microarrays results, we selected representative mRNAs, microRNAs and lncRNAs for validating. For real-time PCR, 7500 Real-Time PCR System (ABI) and SYBR Green Master (Takara) were used. Each sample and negative controls had at least three technical replicates. GAPDH, β-actin and 5S rRNA were amplified under the same conditions as internal controls. The relatives fold change was calculated based on the -ΔΔct method [36].

### Construction of TF (transcription factor)-microRNA-mRNA regulatory loops

TF-microRNA-mRNA loops, representing putative regulatory mechanisms, were constructed based on the microRNA target prediction and functional study database (miRDB) and ChIPBase. We first used ChIPBase to construct TF-microRNA regulatory networks [37]. Considering differentially expressed microRNAs in IBV stimulated DCs, we defined the microRNAs promoter region from the 5 kb upstream to 1 kb downstream region. Then, we extracted TF and microRNA target genes information from ChIPBase. Moreover, we constructed TF-microRNA-mRNAs regulation loops and visualized with cytoscape.

### Statistical analyses

All our data are expressed as the mean ± standard error. Statistical analyses were performed using GraphPad Prism 5 software. Pairwise comparisons were performed using an unpaired two-tailed Student t test. Multiple groups were compared by one-way ANOVA followed with Tukey’s multiple comparison tests. *P* values less than 0.05 were considered to be statistically significant. FlowJo software was selected for the analyzing of our FACS data.

## Results

### Activation of avian or mouse bone marrow-derived dendritic cells (BMDCs) through IBV stimulation

**Fig. 1:**
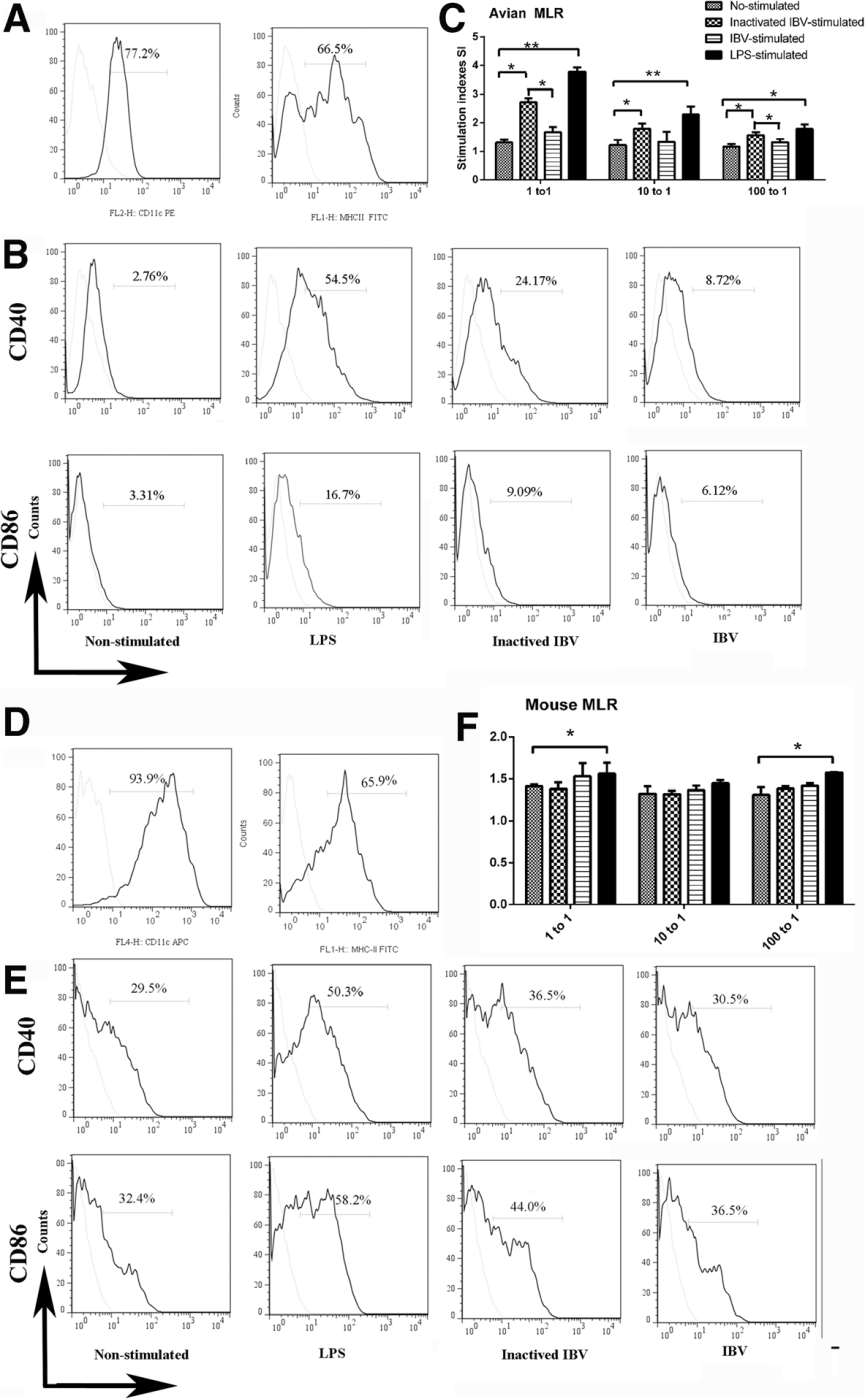
Phenotypic alterations and mixed-lymphocyte reactions (MLR) of avian and mouse BMDCs in response to avian infectious bronchitis virus. **a** Flow cytometric analysis of the phenotypic alterations of immature avian BMDCs at day 7 showed high levels of CD11c and MHC Class II (Black lines display staining with the indicated antibodies, and grey lines the isotype controls).**b** CD40 and CD86 expression of avian BMDCs stimulated by IBV and inactivated IBV (Non-stimulated: immature avian DCs treatment with PBS; LPS: Positive control, immature avian DCs treated with 1 μg/ml LPS; IBV: immature avian DCs stimulated with IBV (MOI = 1); inactivated IBV: immature avian DCs stimulated with inactivated IBV). Shown were representative results of three independent experiments. **c** IBV or inactivated IBV-stimulated avian BMDCs induced the proliferation of lymphocyte cells in MLR. The stimulator cells were BMDCs stimulated with PBS, LPS, IBV or inactivated IBV at 37 °C for 24 h. All experiments were performed at least in triplicate. Significant differences between the treated and Non-stimulated groups are expressed as *P < 0.05 or **P < 0.01, respectively. The significance of the data was determined by one-way ANOVA with Tukey’s multiple comparison test. **d** Flow cytometric analysis of the phenotypic alterations of immature mouse BMDCs showed high levels of CD11c and MHC Class II. **e** Second and third lines: CD40 and CD86 expression of mouse BMDCs stimulated by IBV and inactivated IBV. **f** IBV or inactivated IBV-stimulated mouse BMDCs induced the proliferation of lymphocyte cells in MLR

We investigated how IBV stimulation influences the phenotypic alteration and T lymphocyte activation of avian or mouse BMDCs. Initially, we examined phenotypic changes [38] in avian and mouse DCs stimulated with IBV or inactivated IBV. The fluorescence-activated cell sorting (FACS) results suggested that immature avian BMDCs expressed high levels of cell surface MHC-II (66.5%) and putative CD11c molecules (77.2%), as did immature mouse BMDCs (Fig. 1a). After IBV stimulation, the surface markers CD40 and CD86 were slightly enhanced in avian BMDCs (Fig. 1b) but did not show a notable increase in mouse BMDCs (Fig. 1e). In contrast, the percentages of CD40 and CD86 in inactivated IBV-stimulated avian BMDCs were significantly higher than those in the IBV-stimulated group (Fig. 1b). Interestingly, the CD40 percentage showed no increase in mouse BMDCs stimulated with IBV (Fig. 1e).

Next, we assessed the ability of avian or mouse BMDCs to stimulate T lymphocytes. As shown in Fig. 1c, inactivated IBV-stimulated avian BMDCs exhibited a significant stimulatory capacity in a mixed lymphocyte reaction (MLR) (*P* < 0.01 or *P* < 0.05) compared to naive or IBV-stimulated avian BMDCs. The maximum response was obtained at a ratio of 1:1 between lymphocytes and BMDCs. However, the IBV-stimulated avian BMDCs did not exhibit enhanced ability to activate T lymphocytes compared with inactivated IBV-stimulated avian BMDCs. Interestingly, the performance in activating T lymphocytes was out of the standard range for both IBV-stimulated and inactivated IBV-stimulated mouse BMDCs.

### Identification and bioinformatic analysis of differentially expressed mRNAs

**Fig. 2:**
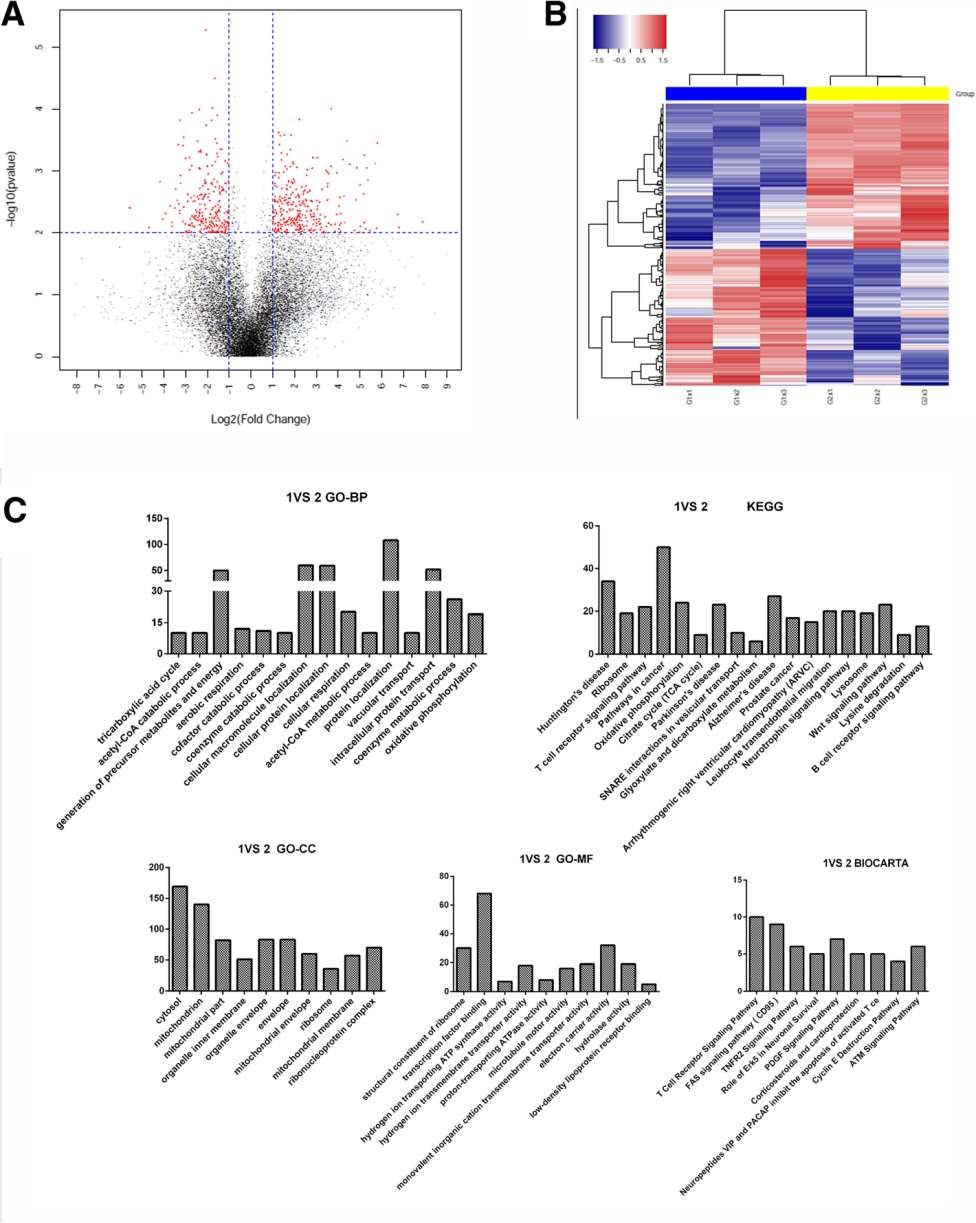
mRNAs microarray analyses of IBV-stimulated avian BMDCs. **a** Volcano plot map of mRNA expression in control and IBV-stimulated avian BMDCs at 12 h post-infection. A comparison of expression data was performed using an XY-scatter plot analysis of the log base two-fold change. Data points shown in red represent significant differentially expressed genes; *P* < 0.01. **b** Heat map of differentially expressed mRNAs in IBV-stimulated avian BMDCs. All of the biological replicates were pooled and calculated to identify differentially expressed mRNAs based on a threshold fold change > 2 and *P* < 0.01. The blue and the yellow group represent two groups. The blue one represent control group, with G1 × 1, G1 × 2, G1 × 3 at the bottom. Whilst the yellow one represent IBV-infected DCs, with G2 × 1, G2 × 2, G2 × 3 at the bottom. **c** Primary GO categorisation and KEGG pathway analyses based on differentially expressed mRNAs in IBV-stimulated avian BMDCs

To clarify the potential roles of host factors involved in IBV infection, mRNA expression of avian BMDCs was examined after stimulation with IBV for 12 h. Based on the criteria of at least a two-fold change and a *P* value less than 0.01, we identified 293 up-regulated and 251 down-regulated genes in IBV-stimulated avian BMDCs (Fig. 2a, b and Additional file 1). Moreover, Gene Ontology (GO) categorisation and pathway analyses of differentially expressed (DE) genes were performed. Cellular macromolecules, the tricarboxylic acid cycle, protein location, and oxidative phosphorylation contributed to the GO biological processes (GO-BP) associated with IBV stimulation (Fig. 2c and Additional file 2). Meanwhile, GO molecular function (GO-MF) and GO cellular component (GO-CC) analyses identified cytosol and transcription factor binding, respectively. We performed pathway analysis using the Kyoto Encyclopedia of Genes and Genomes (KEGG) and BioCarta databases. KEGG analysis showed that genes are significantly differentially regulated in the presence of IBV compared with those in control cells; this is particularly pronounced for oxidative phosphorylation and the T cell receptor and B cell receptor signalling pathways. Based on the BioCarta database, IBV-influenced genes were involved in T cell receptor signalling, FAS signalling, and TNFR2 signalling and played the role of Erk5 in neuronal survival (Fig. 2c and Additional file 2).

### Identification and bioinformatic analysis of differentially expressed miRNAs and their target genes

**Fig. 3:**
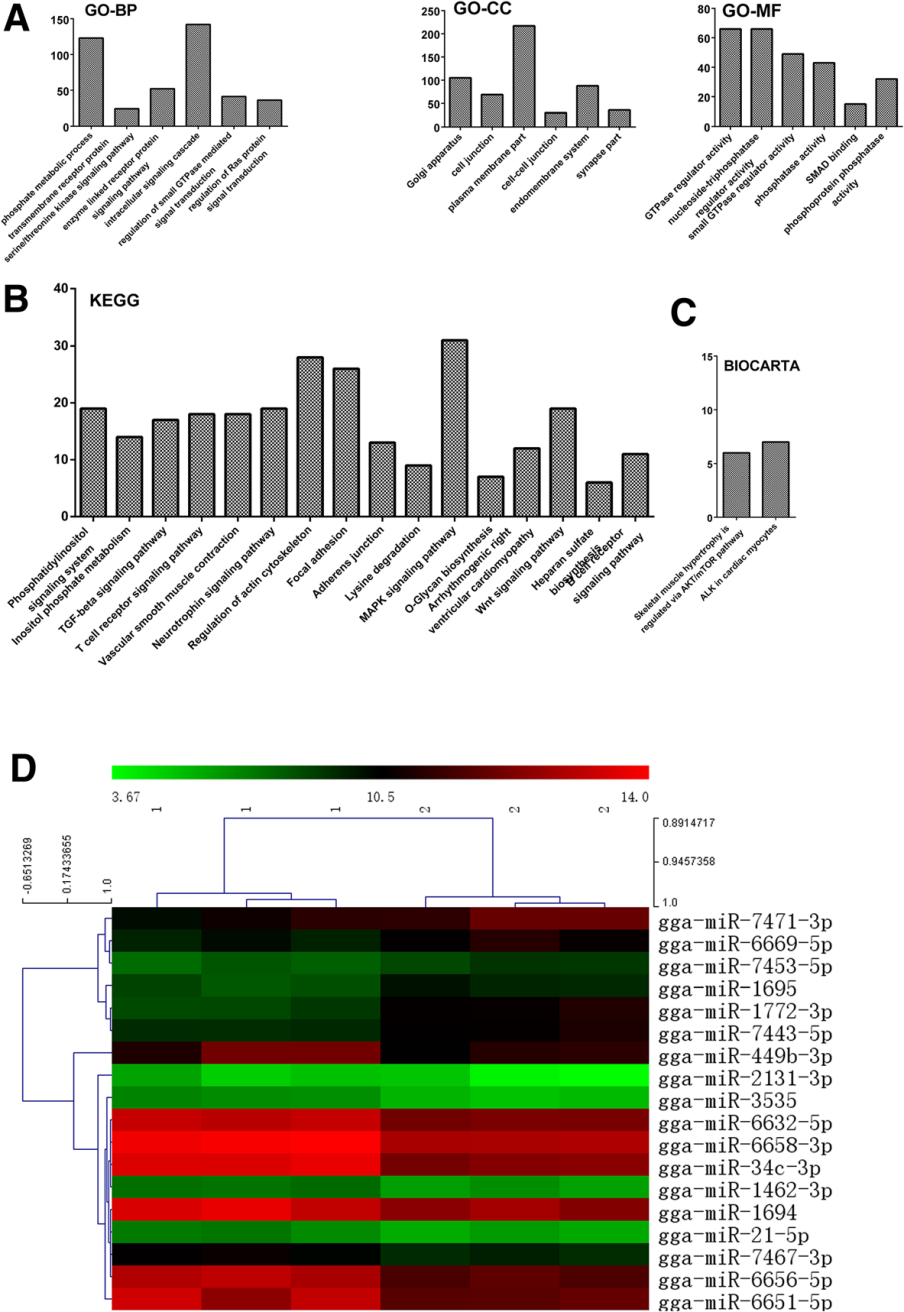
MicroRNAs microarray analyses of IBV-stimulated avian BMDCs. **a** Primary GO categorisation based on target genes from differentially expressed microRNAs in IBV-stimulated avian BMDCs. **b** KEGG pathway analyses based on target genes from differentially expressed microRNAs in IBV-stimulated avian BMDCs. **c** BIOCATRA pathway analyses based on target genes from differentially expressed microRNAs in IBV-stimulated avian BMDCs. **d** Heat map of differentially expressed microRNAs in IBV-stimulated avian DCs. All of the biological replicates were pooled and calculated to identify differentially expressed microRNAs based on a threshold fold change > 2 and *P* < 0.05. The number 1 in the top represent control group, while the number 2 in the top represent IBV-infected BMDCs

Identification of avian microRNA mechanisms could provide an alternative approach to inhibiting viral infection. Therefore, we studied the influence of IBV infection on global miRNA expression in avian BMDCs. First, we detected 991 conserved microRNAs and identified 19 significantly changed miRNAs (12 down-regulated and 7 up-regulated) in IBV-stimulated avian BMDCs using the criteria of a two-fold or greater change and a *P*-value < 0.05 (Fig. 3d and Additional file 3). As we know, miRNAs may function by directly silencing or indirectly reducing their target genes. We predicted the potential targets of DE miRNAs using the MicroRNA Target Prediction and Functional Study Database (miRDB) and TargetScan. We forecasted 124 target genes as DE miRNAs based on the predictions of both programs (Additional file 4). To gain insight into their functions, GO annotation of these target genes was performed; it identified the intracellular signalling cascade and phosphorus metabolic processes in GO-BP categories, whereas GO-MF included GTPase regulator activity and small GTPase regulator activity (Fig. 3a). Finally, KEGG pathway analysis based on the predicted target genes indicated that B cell receptors, regulation of the actin cytoskeleton, and MAPK signalling contributed to miRNA target genes with IBV stimulation (Fig. 3b and Additional file 5). Based on the BioCarta database, the primary pathways associated with IBV infection involved skeletal muscle hypertrophy regulated by AKT/mTOR and ALK in cardiac myocytes (Fig. 3c). The results described above indicated that DE miRNAs play crucial roles in the response to IBV stimulation.

### Identification and bioinformatic analysis of differentially expressed lncRNAs and their targets

**Fig. 4:**
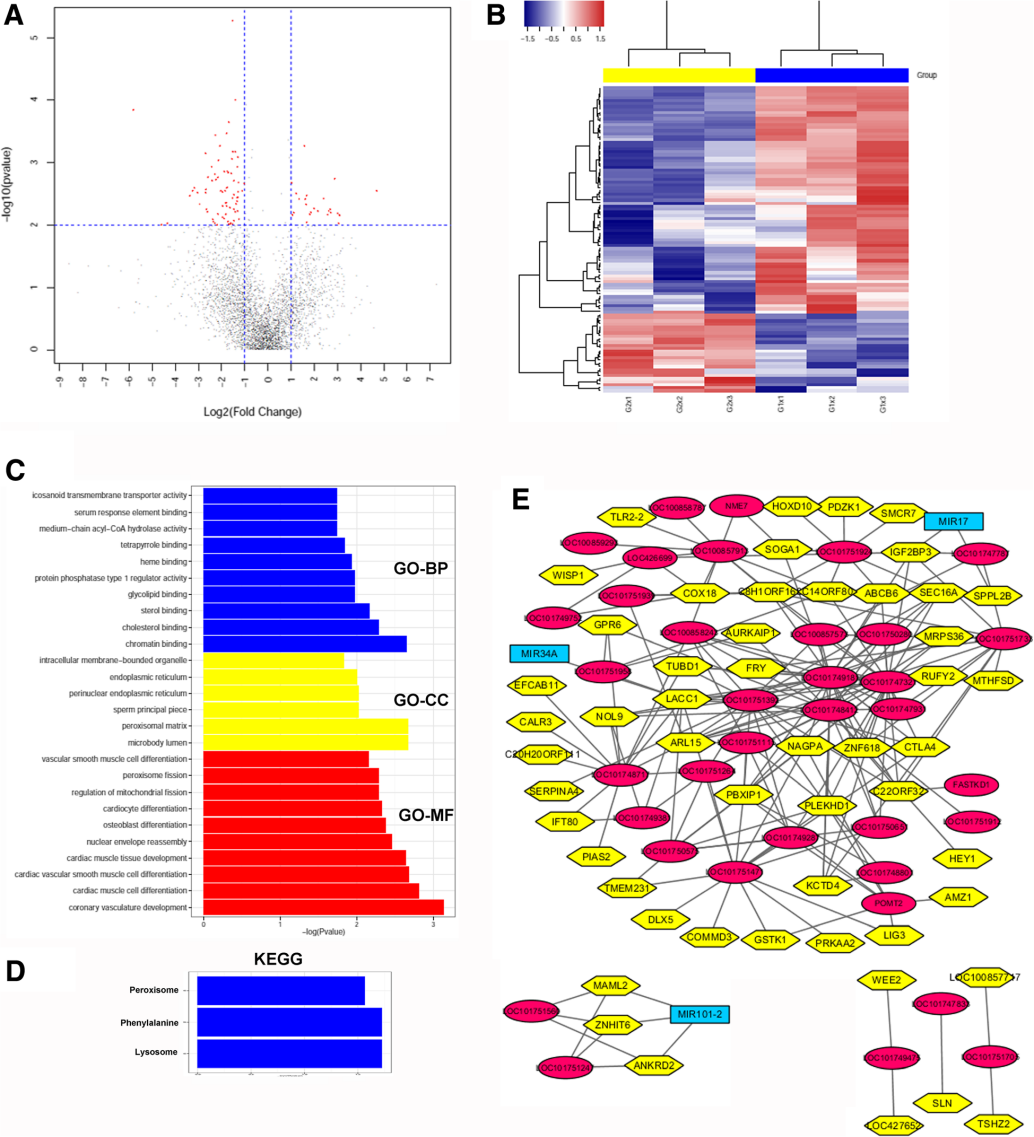
LncRNA microarray analysis of IBV-stimulated avian BMDCs. **a** Volcano plot map of lncRNA expression levels in control avian BMDCs and IBV-stimulated avian BMDCs at 12 h post-infection. A comparison of expression data was performed using XY-scatter plot analysis of the log base two-fold change. Data points shown in red represent significantly differentially expressed genes; *P* < 0.01. **b** Heat map of differentially expressed lncRNAs in IBV-stimulated avian BMDCs. All biological replicates were pooled to identify differentially expressed lncRNAs based on a threshold fold change > 2 and *P* < 0.01. The blue and yellow colours represent the two groups, with blue showing the control group, with G1 × 1, G1 × 2, G1 × 3 at the bottom, whereas yellow represents IBV-infected DCs, with G2 × 1, G2 × 2, G2 × 3 at the bottom.**c** Primary GO categorisation based on *cis*-, *trans*-, and co-expressed target genes of differentially expressed lncRNAs in IBV-stimulated avian BMDCs.**d** KEGG pathway analysis based on target genes of differentially expressed lncRNAs in IBV-stimulated avian BMDCs. **e** Potential interaction network among significantly differentially expressed lncRNAs and target genes, created using Cytoscape (The pink ellipse represents co-expressed lncRNAs, while the yellow hexagon represents co-expressed mRNAs). All biological replicates were pooled to identify differentially expressed lncRNAs based on a threshold fold change > 2 and *P* < 0.01

Long non-coding RNAs (lncRNAs) have recently been identified as regulators of many biological processes, especially in the immune response. Global lncRNA expression levels were evaluated in avian BMDCs stimulated with IBV, and 101 (26 down-regulated, 75 up-regulated) DE lncRNAs were identified among a total of 3900 lncRNAs (Fig. 4a, b, and Additional file 5). Because lncRNAs function through *cis-, trans-*, or co-expression with target genes, we then predicted the potential *cis, trans* and co-expressed genes among the DE lncRNAs. We identified 30 *cis* and 3941 *trans* targets, as well as 203 co-expressed mRNAs (Additional file 6). The interactions among co-expressed lncRNAs and mRNAs were mapped and are presented in Fig. 4e. Further analyses of the GO categorisation and KEGG pathways were performed based on cis and trans target to illustrate the functions of lncRNAs associated with IBV infection. The results of GO-BP analysis showed positive regulation of coronary vasculature development, cardiac muscle development, and nuclear envelope reassembly (Fig. 4c and Additional file 7). KEGG pathway analysis indicated the peroxisome, phenylalanine, and lysosome signal pathways were associated with IBV infection of avian BMDCs (Fig. 4d and Additional file 7).

### Confirmation of microarray results through qRT-PCR

**Fig. 5:**
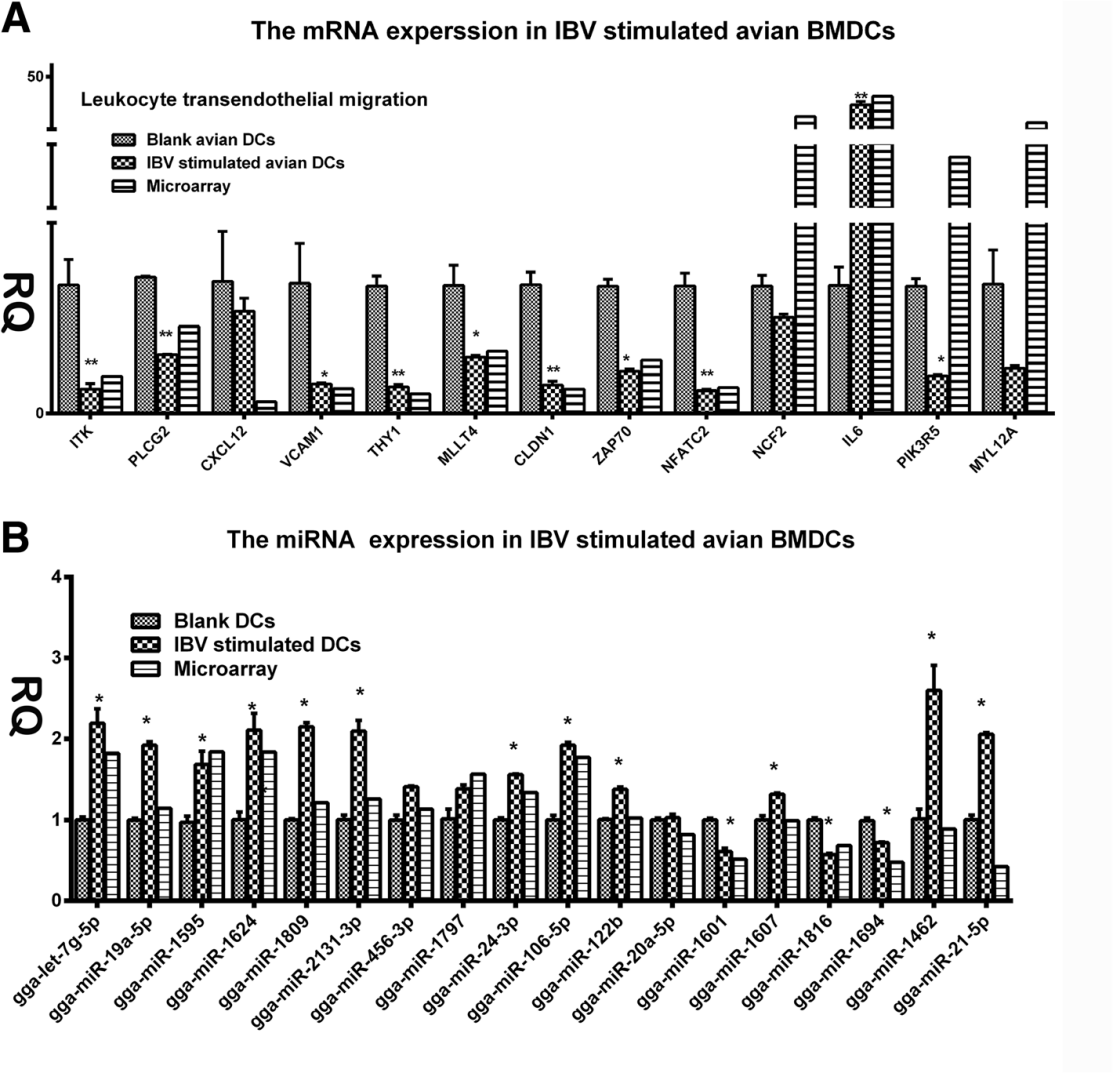
Results of the qPCR analysis of select mRNAs, miRNAs and lncRNAs following stimulation by IBV stimulation (All of the experiments were performed at least in triplicate. Significant differences between the treated and control groups are expressed as *P < 0.05 and **P < 0.01, respectively) **a** Expression levels of mRNAs involved in the leukocyte trans-endothelial migration signal pathway. **b** Expression levels of microRNAs qPCR results of the significantly up-regulated or down-regulated microRNAs in the IBV-stimulated group

To validate the mRNA, microRNA, and lncRNA microarray results, we subjected 13 DE mRNAs and 18 DE miRNAs to quantitative reverse transcription polymerase chain reaction (qRT-PCR) (Tables 1 and 2). Generally, the qRT-PCR data matched the microarray data, with fold-change values of representative mRNAs and miRNAs in qRT-PCR displaying trends similar to those reflected in the results of microarray analysis. On one hand, the 13 selected mRNAs were primarily associated with leucocyte transendothelial migration signalling pathways. In this pathway, ITK, PLCG2, CXCL12, VCAM1, CLDN1, ZAP70, THY1, and MLLT4 expression levels significantly decreased with IBV stimulation, whereas only IL-6 expression significantly increased in IBV-infected avian BMDCs (Fig. 5a). On the other hand, among the 18 tested microRNAs, gga-miRlet7g, gga-miR19a, gga-miR1595, gga-miR1624, gga-miR1809, gga-miR2131, gga-miR24, gga-miR106, gga-miR122b, gga-miR1607, gga-miR1462, and gga-miR21 were significantly up-regulated when stimulated with IBV. In contrast, gga-miR1601, gga-miR1816, and gga-miR1694 were significantly down-regulated after IBV stimulation (Fig. 5b).

**Table 1:** QRT-PCR primers used in verification of microarrays results

Gene	Sence	Anti-sence
GAPDH	TGACCACTGTCCATGCCATC	CAGCAGCCTTCACTACCCTC
Leukocyte transendothelial migration		
ITK	tggaagaagaaggccccaat	catcgctccttcacgaatgg
NFATC2	cagcatcaaaccccatcgag	atctgctgcccatctgaagt
PIK3R5	gcacttcctaccattgcagg	tcctcctcctcctcttcctc
PLCG2	gctttgtggctctcagatgg	agcttagggagatgacgagc
CXCL12	gatgcccctgtcgattcttc	tcctggatccattttagcttgg
ZAP70	tatggagctacggtgtgacc	aggtgcggattgtgttttcc
MYL12A	caatggcactgatccggaag	tccatgtttaaggatgcgcg
CLDN1	ctgtctttggtggcgtgatc	taggatgtttcactccgggg
VCAM1	gagaaaccgccactgtcatc	tctggccacacaaacaatctc
MLLT4	taattcctccccagcctgtg	gtactgcagatctctccgct
THY1	ccaaggacaacaggaagcac	ccttcagctcgcacatgtag
IL6	acgtcgagtctctgtgctac	ggcactgaaactcctggtct
NCF2	cacggagggagagggatttt	cattgcccttccaaccagtc

**Table 2:** QRT-PCR primers used in verification of avian microRNAs MicroRNA array results

MiRNAs	Sence primer
gga-miR-2131-3p	GGTGCTGTTACTGTTCTTCTGATGG
gga-miR-1462-3p	GTATCTGTCCTTGTGAGCCCCAG
gga-miR-21-5p	GCGTAGCTTATCAGACTGATGTTGA
gga-miR-1694	TAATAAAGGAGGACGAGGCTGCGAGC
gga-miR-1607	AATTAATTATAGGGGCGGGAGGGGTCG
gga-let-7 g-5p	TGGGTGGGTGAGGTAGTAGTTTGTACAG
gga-miR-19a-5p	GCGAGTTTTGCATAGTTGCACTAC
gga-miR-1797	GCTTGGAACTGAGCAGGAACTG
gga-miR-20a-5p	CGGTAAAGTGCTTATAGTGCAGGTAG
gga-miR-106-5p	CGGAAAAGTGCTTACAGTGCAGGT
gga-miR-1595-5p	AAGCACGAGTGTGGTGGAGCTC
gga-miR-122b	CGGAGTGTGACACTGGTGTTTTT
gga-miR-1624	TAATACACCGCACTGGCAGGGA
gga-miR-1809	GTGGGAAGTTTGGCAGAGCAT
gga-miR-24-3p	TGGCTCAGTTCAGCAGGAACAG
gga-miR-1816	GTGGGTAGGTTTTGTGGTTTTGTT
gga-miR-456-3p	GGCAGGCTGGTTAGATGGTTGTC
gga-miR-1601	AGTGTGAGCAGGTGCAGAGCTG

### Establishment of TF–miRNA–mRNA regulatory loops

**Fig. 6:**
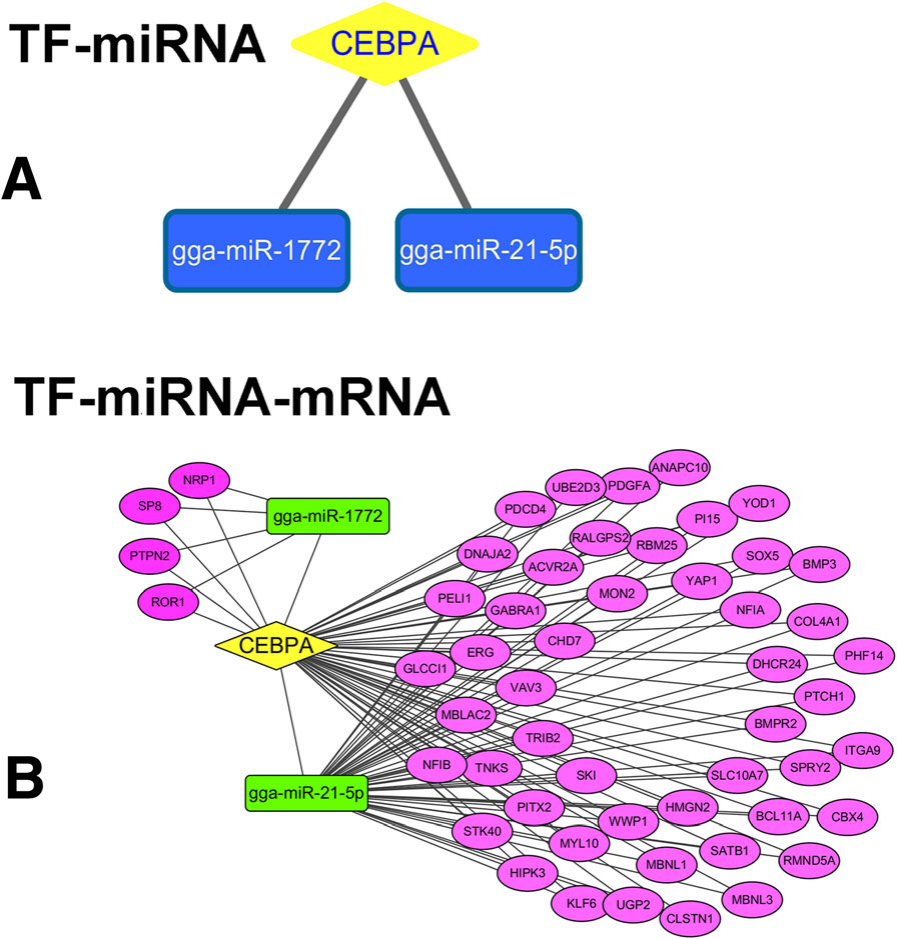
TF–miRNA and TF–miRNA–mRNA regulatory loops in IBV-stimulated avian BMDCs. **a** Two TF-miRNA networks involving one TF and two differentially expressed microRNAs were established, with the TF CEBPA binding directly to the promoters of gga-miR1772 and gga-miR21. **b** In total, 53 TF-miRNA-mRNA interactions involving 1 TF (CEBPA), 2 differentially expressed microRNAs, and 53 differentially expressed mRNAs (and predicted microRNA targets) for the IBV-stimulated group were summarised. Yellow diamond nodes represent TFs, green rectangle nodes correspond to microRNAs, and violet ovals represent mRNAs

To better understand how IBV regulates gene expression in avian BMDCs, we constructed transcription factor (TF)–miRNAs and TF–miRNA–mRNA regulatory loops. First, we computed TF-microRNA loops based on 165 TF-microRNA combinations involving 2 TFs and 149 microRNAs (Additional file 8). All of the predicted TF-miRNA regulation loops still need for further validation. Considering the DE miRNAs, we identified two TF-microRNA networks (CCAAT/enhancer-binding protein alpha [CEBPA]-gga-miR-1772 and CEBPA-gga-miR-21-5p) in the IBV-stimulated avian BMDCs (Fig. 6a). Then, we extracted and constructed the TF and mRNA relationship data from ChIPBase. In total, 21,085 TF-mRNAs pairs were computed, involving 2 TFs and 5987 mRNAs (Additional file 9). Finally, TF-mRNA and miRNA-target mRNA information were combined to produce TF-miRNA-mRNA regulatory networks (Fig. 6b). In total, we identified 53 TF–miRNA–mRNA interactions, which involved 1 TF, 2 miRNAs, and 53 mRNAs in IBV-stimulated avian DCs. For example, ROR1, NRP1, S8, and PTPN2 could be regulated by both CEBPA and miR-1772, whereas miR-1772 could also be modulated by CEBPA. Thus, we constructed the network to link CEBPA-miR-1772-ROR1 (Fig. 6b, Additional file 10).

## Discussion

Avian infectious bronchitis, caused by infectious bronchitis virus, has been identified as an important pathogen in poultry that is characterised by severity and outcomes that make it extremely difficult to control [1, 39, 40]. IBV primarily replicates in the epithelial cells of the respiratory tract and can affect both meat-type and commercial egg-laying birds [1, 5]. In this study, host responses against IBV were analysed using microarrays of mRNAs, miRNAs, and lncRNAs. Our study provided information about the molecular pathogenesis and virus–host interactions between IBV and avian BMDCs. Further bioinformatic analysis revealed that the leukocyte transendothelial migration signal pathway might be involved in IBV-infected avian BMDCs. Information obtained herein should provide useful clues for the development of novel preventive or therapeutic strategies against IBV infection.

### Phenotypic alteration and lymphocyte activation of avian DCs stimulated with IBV

The strategy of controlling IBV through vaccination has been partially successful, but various subtypes of IBV still evade vaccines [40]. DCs change during the maintenance of immune responses, and their maturation is pivotal to the development of immunity against many viruses. The maturation of avian BMDCs is essential to the development of the immune system. Viruses, therefore, have evolved strategies to target this process [41, 42]. In our studies, IBV-stimulated DCs (including mouse and avian DCs) did not significantly increase their expression of the surface markers CD40 and CD86, indicating that IBV might escape recognition by avian DCs. This phenomenon might be attributed to failed activation of toll-like receptor (TLR) signalling, which is related to the recognition of various dangerous signals [43, 44]. Previous studies have suggested that the binding of IBV to epithelial cells initiates infection [45]. Recent studies suggest that the binding progress is sialic acid-dependent and that chicken homologues of the human receptors DC-SIGN and L-SIGN are part of an IBV receptor complex [46–48]. In our study, mRNA microarray results indicated that TLR7, TLR3, and TLR21 were all down-regulated in IBV-stimulated avian DCs. The decreased expression of TLR3 suggested that IBV might be unsuccessfully replicated in avian DCs (Additional file 11). Receptors, such as TLR3 and TLR7, may initiate the local tracheal innate immune response upon IBV recognition [49, 50]. The accordance of TLR results with surface marker results suggest that IBV successfully escaped recognition by avian DCs. A key role of DCs is to process and present specific antigens to naïve T cells [51–53]. Consistent with the surface marker results, cell proliferation studies found that both IBV-treated avian and mouse DCs failed to stimulate the proliferation of naïve T cells in MLR [54, 55]. In contrast, inactivated IBV strongly activated the primary T cell, which was correlated with greater expression of the co-stimulatory molecules CD40 and CD86 by inactivated IBV-stimulated DCs. Recent study indicating that host shutoff by IBV plays an important role in antagonizing the host’s innate immune response and demonstrate that 5b is a functional equivalent of nsp1, charge for the host shutoff. These results show that avian DCs react more strongly to inactivated IBV than to IBV in terms of enhanced maturation and ability to activate lymphocytes.

### Roles of microRNAs and lncRNAs during IBV infection

Aside from direct gene regulation, miRNAs and lncRNAs also play important roles in regulating the function of DCs. Alteration of cellular microRNA expression levels during viral infection is a component of the host–virus interaction [56]. Viruses not only influence the expression of microRNAs in infected cells but also encode viral small RNAs that regulate the replication of viruses, as reported for PRRSV and Dengue virus [57, 58]. Host microRNAs, such as avian miRNAs, might enhance the immune response or be utilised by viruses to assist with their replication. For example, miR140 is a post-transcriptional regulator influencing the expression of SPP1, which plays important roles in the inflammatory response, calcification, organ development, and immune cell function [59, 60]. Cellular microRNAs (miR-323, miR-491, and miR-654) have also been found to regulate the replication of H1N1 influenza virus by targeting the PB1 gene [61].

In this study, we attempted to determine how miRNAs and lncRNAs, induced by IBV infection, regulate the maturation and antigen presentation of avian DCs. Initially, 991 chicken microRNAs were investigated, and 19 differentially expressed microRNAs were identified in IBV-infected avian DCs. Among these miRNAs, gga-miR-135A, gga-miR-7471, gga-miR-7453, gga-miR-7443, gga-miR-1695, gga-miR-1772, and gga-miR-6669 were significantly up-regulated in IBV-infected avian BMDCs, whereas 12 other gga-microRNAs were down-regulated. Among the microRNAs that decreased, miR-21 was associated with lymphoma development, which might be a possible mechanism of lymphocyte activation [62, 63]. Recent studies have reported the functions of many gga-miRNAs, including gga-miR-1454, gga-miR-215-5p, gga-miR-3538, gga-miR-2954, and gga-miR-1a-3p [20, 64, 65]. One of these studies suggested that gga-miR-215-5p exhibited significantly varied expression levels between H9N2-infected and non-infected chicken embryo fibroblasts [65]. Further bioinformatic analysis to identify the target genes of gga-microRNAs revealed that IBV infection not only impacted the TGF-beta and MAPK signalling pathways but also influenced the T cell receptor and B cell receptor signalling pathways (Fig. 3). Previous research suggested that the complement system was important in T cell activation, as it triggers the antiviral state of neighbouring cells and an influx of T cells to the local tissue [66]. Several studies have shown that the major histocompatibility complex (MHC) influences susceptibility to IBV infection [67, 68]. Meanwhile, lncRNAs are involved in defending against viral infection. Transcriptional regulation by lncRNAs can occur in either a *cis-* or t*rans-* manner. Of the down-regulated lncRNAs, MANBAL and POMT2 were correlated with mannose, which might be a receptor for viral entry [69, 70]. Mannose-binding lectin (MBL) is involved in the protection of the host against viral infections, such as infections with influenza A virus, hepatitis C virus, and Ebola virus [71–73]. In chickens, MBL serum concentrations have also been associated with IBV infection [4]. Additionally, we found that *cis-* and *trans-*targeting genes contribute to the peroxisome, phenylalanine, and lysosome signalling pathways.

### TF-miRNA-mRNA regulatory networks in IBV-stimulated DCs

TF-microRNA networks have been previously identified as important components of microRNA regulation mechanisms. Our study identified two TF-microRNA networks in the IBV-stimulated group (CEBPA-gga-miR1772 and CEBPA-gga-miR21). CEBPA is a transcription factor involved in the differentiation of certain blood cells. The encoded protein can interact with CDK2 and CDK4, thereby inhibiting these kinases and causing arrest of growth in cultured cells. Additionally, CEBPA is essential for myeloid lineage commitment and is therefore required for both normal mature granulocyte formation and the development of abnormal acute myeloid leukaemia (AML) [74, 75]. On the other hand, gga-miR-21 was suggested to inhibit chicken pre-adipocyte proliferation by down-regulating Kruppel-like factor 5 [72]. This suggests that gga-miR-21 can be regulated as a defence mechanism to fight against viral infection in birds. To further explore the mechanisms involved, we construct TF-microRNA-mRNA networks, which provided insights into the interplays between microRNAs and TF and hinted at the mechanisms underlying the innate cellular response induced by IBV stimulation.

## Conclusions

In this study, microarray analysis of avian DCs stimulated with IBV provided insight into the mechanisms underlying the innate cellular response induced by IBV. Specifically, the leukocyte transendothelial migration signalling pathway was found to be involved in the regulation of avian DCs stimulated with IBV. Two TF–miRNA networks (CEBPA-gga-miR1772 and CEBPA-gga-miR21) and 53 TF–miRNA–mRNA networks were identified in IBV-stimulated DCs, elucidating host antiviral defences and offering novel preventive strategies against IBV.

## Additional files

Additional file 1: Differentially expressed mRNAs in IBV-stimulated avian BMDCs. (XLS 9410 kb)

Additional file 2: GO and KEEG analysis of differentially expressed mRNAs in IBV-stimulated avian BMDCs. (XLS 208 kb)

Additional file 3: Differentially expressed miRNAs in IBV-stimulated avian BMDCs. (XLS 394 kb)

Additional file 4: The target gene of differentially expressed miRNAs in IBV-stimulated avian BMDCs and their GO and KEEG analysis. (XLS 223 kb)

Additional file 5: Differentially expressed LncRNAs in IBV-stimulated avian BMDCs. (XLSX 30 kb)

Additional file 6: The cis and trans target gene of differentially expressed lncRNAs in IBV-stimulated avian BMDCs. (XLS 517 kb)

Additional file 7: GO and KEEG analysis of differentially expressed lncRNAs target in IBV-stimulated avian BMDCs. (XLS 458 kb)

Additional file 8: microRNA and TF invloved in TF-microRNA loops. (TXT 21 kb)

Additional file 9: TF and mRNA invloved in TF-mRNAs pairs. (XLS 902 kb)

Additional file 10: Selected TF-miRNA-mRNA loops. (XLS 22 kb)

Additional file 11: TLR family invloved in IBV-stimulated avian BMDCs. (XLSX 10 kb)

## Data Availability

The authors had to send and upload the microarrays data into the Gene Expression Omnibus (GEO; https://www.ncbi.nlm.nih.gov/geo/query/acc.cgi?acc=GSE100802.) and has been assigned provisional series accession GSE100802.

## Abbreviations

3’UTR: Three prime untranslated region
BMDCs: Bone marrow dendritic cells
BP: Biological process
CC: Cellular component
DAVID: The Database for Annotation, Visualization and Integrated Discovery
DCs: Dendritic cells
FACS: Fluorescence Activated Cell Sorter
GM-CSF: Granulocyte colony-stimulating factor
GO: Gene ontology
IBV: Infectious bronchitis virus
JAAS: Jiangsu Academy of Agricultural Sciences
KEGG: Kyoto Encyclopedia of Genes and Genomes
lncRNA: Long non-coding RNA
MAPK: Mitogen-activated protein kinases
MHC-II: Major histocompatibility complex class II
TF: Transcription factor

## References

[1] Bande F, Arshad SS, Omar AR, Hair-Bejo M, Mahmuda A, Nair V (2017). Global distributions and strain diversity of avian infectious bronchitis virus: a review. Anim Health Res Rev.

[2] Marandino A, Tomas G, Panzera Y, Greif G, Parodi-Talice A, Hernandez M, Techera C, Hernandez D, Perez R (2017). Whole-genome characterization of Uruguayan strains of avian infectious bronchitis virus reveals extensive recombination between the two major south American lineages. Infect, Genet Evol.

[3] Smith J, Sadeyen JR, Cavanagh D, Kaiser P, Burt DW (2015). The early immune response to infection of chickens with infectious bronchitis virus (IBV) in susceptible and resistant birds. BMC Vet Res.

[4] Kjaerup RM, Dalgaard TS, Norup LR, Hamzic E, Sorensen P, Juul-Madsen HR (2014). Characterization of cellular and humoral immune responses after IBV infection in chicken lines differing in MBL serum concentration. Viral Immunol.

[5] Britton P, Armesto M, Cavanagh D, Keep S (2012). Modification of the avian coronavirus infectious bronchitis virus for vaccine development. Bioengineered Bugs.

[6] Habibi M, Karimi V, Langeroudi AG, Ghafouri SA, Hashemzadeh M, Farahani RK, Maghsoudloo H, Abdollahi H, Seifouri P (2017). Combination of H120 and 1/96 avian infectious bronchitis virus vaccine strains protect chickens against challenge with IS/1494/06 (variant 2)-like infectious bronchitis virus. Acta Virol.

[7] Ndegwa EN, Toro H, van Santen VL (2014). Comparison of vaccine subpopulation selection, viral loads, vaccine virus persistence in trachea and cloaca, and mucosal antibody responses after vaccination with two different Arkansas Delmarva poultry industry -derived infectious bronchitis virus vaccines. Avian Dis.

[8] Okino CH, Alessi AC, Montassier Mde F, Rosa AJ, Wang X, Montassier HJ (2013). Humoral and cell-mediated immune responses to different doses of attenuated vaccine against avian infectious bronchitis virus. Viral Immunol.

[9] Ambali AG, Jones RC (1990). Early pathogenesis in chicks of infection with an enterotropic strain of infectious bronchitis virus. Avian Dis.

[10] Cavanagh D, Casais R, Armesto M, Hodgson T, Izadkhasti S, Davies M, Lin F, Tarpey I, Britton P (2007). Manipulation of the infectious bronchitis coronavirus genome for vaccine development and analysis of the accessory proteins. Vaccine.

[11] Juul-Madsen HR, Norup LR, Jorgensen PH, Handberg KJ, Wattrang E, Dalgaard TS (2011). Crosstalk between innate and adaptive immune responses to infectious bronchitis virus after vaccination and challenge of chickens varying in serum mannose-binding lectin concentrations. Vaccine.

[12] Jiang L, Zhao W, Han Z, Chen Y, Zhao Y, Sun J, Li H, Shao Y, Liu L, Liu S (2017). Genome characterization, antigenicity and pathogenicity of a novel infectious bronchitis virus type isolated from South China. Infect Genet Evol.

[13] Zhou S, Tang M, Jiang Y, Chen X, Shen X, Li J, Dai Y, Zou J (2014). Complete genome sequence of a novel infectious bronchitis virus strain circulating in China with a distinct S gene. Virus Genes.

[14] Amarasinghe A, Abdul-Cader MS, Nazir S, De Silva SU, van der Meer F, Cork SC, Gomis S, Abdul-Careem MF (2017). Infectious bronchitis corona virus establishes productive infection in avian macrophages interfering with selected antimicrobial functions. PLoS One.

[15] Ammayappan A, Vakharia VN (2009). Complete nucleotide analysis of the structural genome of the infectious bronchitis virus strain md27 reveals its mosaic nature. Viruses.

[16] Alonso-Caplen FV, Matsuoka Y, Wilcox GE, Compans RW (1984). Replication and morphogenesis of avian coronavirus in Vero cells and their inhibition by monensin. Virus Res.

[17] Wang Y, Qi X, Gao H, Gao Y, Lin H, Song X, Pei L, Wang X (2009). Comparative study of the replication of infectious bursal disease virus in DF-1 cell line and chicken embryo fibroblasts evaluated by a new real-time RT-PCR. J Virol Methods.

[18] Jauregui-Zuniga D, Pedraza-Escalona M, Espino-Solis GP, Quintero-Hernandez V, Olvera-Rodriguez A, Diaz-Salinas MA, Lopez S, Possani LD (2017). Targeting antigens to Dec-205 on dendritic cells induces a higher immune response in chickens: hemagglutinin of avian influenza virus example. Res Vet Sci.

[19] Nagy N, Bodi I, Olah I (2016). Avian dendritic cells: phenotype and ontogeny in lymphoid organs. Dev Comp Immunol.

[20] Lin J, Xia J, Zhang K, Yang Q (2016). Genome-wide profiling of chicken dendritic cell response to infectious bursal disease. BMC Genomics.

[21] Seo W, Taniuchi I (2017). Regulation of hematopoiesis and immune responses by long non-coding RNAs. Int Immunol.

[22] Tarifeno-Saldivia E, Valenzuela-Miranda D, Gallardo-Escarate C (2017). In the shadow: the emerging role of long non-coding RNAs in the immune response of Atlantic salmon. Dev Comp Immunol.

[23] He R, Gu X, Lai W, Peng X, Yang G (2017). Transcriptome-microRNA analysis of Sarcoptes scabiei and host immune response. PLoS One.

[24] Robertson Sarah A., Zhang Bihong, Chan Honyueng, Sharkey David J., Barry Simon C., Fullston Tod, Schjenken John E. (2017). MicroRNA regulation of immune events at conception. Molecular Reproduction and Development.

[25] Lin J, Xia J, Chen YT, Zhang KY, Zeng Y, Yang Q (2017). H9N2 avian influenza virus enhances the immune responses of BMDCs by down-regulating miR29c. Vaccine.

[26] Lin J, Xia J, Tu CZ, Zhang KY, Zeng Y, Yang Q (2017). H9N2 avian influenza virus protein PB1 enhances the immune responses of bone marrow-derived dendritic cells by Down-regulating miR375. Front Microbiol.

[27] Rosenberger CM, Podyminogin RL, Diercks AH, Treuting PM, Peschon JJ, Rodriguez D, Gundapuneni M, Weiss MJ, Aderem A (2017). miR-144 attenuates the host response to influenza virus by targeting the TRAF6-IRF7 signaling axis. PLoS Pathog.

[28] Wang Z, Jiang D, Zhang C, Tan H, Li Y, Lv S, Hou X, Cui X (2015). Genome-wide identification of turnip mosaic virus-responsive microRNAs in non-heading Chinese cabbage by high-throughput sequencing. Gene.

[29] Lin J, Chen YT, Xia J, Yang Q (2016). MiR674 inhibits the neuraminidase-stimulated immune response on dendritic cells via down-regulated Mbnl3. Oncotarget.

[30] Lucio B (1990). Fabricant J:tissue tropism of three cloacal isolates and Massachusetts strain of infectious bronchitis virus. Avian Dis.

[31] Fu J, Liang J, Kang HH, Lin J, Yu QH, Yang Q (2014). The stimulatory effect of different CpG oligonucleotides on the maturation of chicken bone marrow-derived dendritic cells. Poult Sci.

[32] Yin Y, Qin T, Wang X, Lin J, Yu Q, Yang Q (2015). CpG DNA assists the whole inactivated H9N2 influenza virus in crossing the intestinal epithelial barriers via transepithelial uptake of dendritic cell dendrites. Mucosal Immunol.

[33] Takashi Y, Tatsuya M (2011). Integration of pre-normalized microarray data using quantile correction. Bioinformation.

[34] Peng Z, Wang J, Shan B, Yuan F, Li B, Dong Y, Peng W, Shi W, Cheng Y, Gao Y, Zhang C (2017). Duan: Genome-wide analyses of long noncoding RNA expression profiles in lung adenocarcinoma. Sci Rep.

[35] Shannon P, Markiel A, Ozier O, Baliga NS, Wang JT, Ramage D, Amin N, Schwikowski B, Ideker T (2003). Cytoscape: a software environment for integrated models of biomolecular interaction networks. Genome Res.

[36] Cyplik P, Schmidt M, Szulc A, Marecik R, Lisiecki P, Heipieper HJ, Owsianiak M, Vainshtein M, Chrzanowski L (2011). Relative quantitative PCR to assess bacterial community dynamics during biodegradation of diesel and biodiesel fuels under various aeration conditions. Bioresour Technol.

[37] Yang JH, Li JH, Jiang S, Zhou H, Qu LH (2013). ChIPBase: a database for decoding the transcriptional regulation of long non-coding RNA and microRNA genes from ChIP-Seq data. Nucleic Acids Res.

[38] Banchereau J, Briere F, Caux C, Davoust J, Lebecque S, Liu YJ, Pulendran B, Palucka K (2000). Immunobiology of dendritic cells. Annu Rev Immunol.

[39] Quinteros JA, Lee SW, Markham PF, Noormohammadi AH, Hartley CA, Legione AR, Coppo MJ, Vaz PK, Browning GF (2016). Full genome analysis of Australian infectious bronchitis viruses suggests frequent recombination events between vaccine strains and multiple phylogenetically distant avian coronaviruses of unknown origin. Vet Microbiol.

[40] Bande F, Arshad SS, Omar AR, Bejo MH, Abubakar MS, Abba Y (2016). Pathogenesis and diagnostic approaches of avian infectious bronchitis. Adv Virol.

[41] Real-Time R (2009). Emergence of a novel swineorigin influenza a (H1N1) virus in humans. N Engl J Med.

[42] Rouse BT, Sehrawat S (2010). Immunity and immunopathology to viruses: what decides the outcome?. Nat Rev Immunol.

[43] Cardinaud S, Urrutia A, Rouers A, Coulon PG, Kervevan J, Richetta C, Bet A, Maze EA, Larsen M, Iglesias MC (2017). Triggering of TLR-3, −4, NOD2, and DC-SIGN reduces viral replication and increases T-cell activation capacity of HIV-infected human dendritic cells. Eur J Immunol.

[44] Palucka K, Banchereau J (2002). How dendritic cells and microbes interact to elicit or subvert protective immune responses. Curr Opin Immunol.

[45] Seifi S, Boroomand Z (2015). Ultrastructural study of the trachea in experimentally infected broilers with IBV serotype 4/91. Slov Vet Res.

[46] Winter C, Herrler G, Neumann U (2008). Infection of the tracheal epithelium by infectious bronchitis virus is sialic acid dependent. Microbes Infect.

[47] Abd El Rahman S, El-Kenawy AA, Neumann U, Herrler G, Winter C (2009). Comparative analysis of the sialic acid binding activity and the tropism for the respiratory epithelium of four different strains of avian infectious bronchitis virus. Avian Pathol.

[48] Zhang Y, Buckles E, Whittaker GR (2012). Expression of the C-type lectins DC-SIGN or L-SIGN alters host cell susceptibility for the avian coronavirus, infectious bronchitis virus. Vet Microbiol.

[49] Guo X, Rosa AJ, Chen DG, Wang X (2008). Molecular mechanisms of primary and secondary mucosal immunity using avian infectious bronchitis virus as a model system. Vet Immunol Immunopathol.

[50] Wang X, Rosa AJ, Oliverira HN, Rosa GJ, Guo X, Travnicek M, Girshick T (2006). Transcriptome of local innate and adaptive immunity during early phase of infectious bronchitis viral infection. Viral Immunol.

[51] Lu Erick, Dang Eric V., McDonald Jeffrey G., Cyster Jason G. (2017). Distinct oxysterol requirements for positioning naïve and activated dendritic cells in the spleen. Science Immunology.

[52] Maggi J, Schinnerling K, Pesce B, Hilkens CM, Catalan D, Aguillon JC (2016). Dexamethasone and Monophosphoryl lipid A-modulated dendritic cells promote antigen-specific Tolerogenic properties on naive and memory CD4+ T cells. Front Immunol.

[53] Sato K, Honda SI, Shibuya A, Shibuya K (2016). Improved protocol for the isolation of naive follicular dendritic cells. Mol Immunol.

[54] Groux H (2004). Role of dendritic cells in the generation of regulatory T cells. Seminars in Immunology.

[55] Beckebaum S, Cicinnati VR, Zhang X, Ferencik S, Frilling A, Grosse-Wilde H, Broelsch CE, Gerken G (2003). Hepatitis B virus-induced defect of monocyte-derived dendritic cells leads to impaired T helper type 1 response in vitro: mechanisms for viral immune escape. Immunology.

[56] Umbach JL, Cullen BR (2009). The role of RNAi and microRNAs in animal virus replication and antiviral immunity. Genes Dev.

[57] Li N, Yan Y, Zhang A, Gao J, Zhang C, Wang X, Hou G, Zhang G, Jia J, Zhou EM (2016). MicroRNA-like viral small RNA from porcine reproductive and respiratory syndrome virus negatively regulates viral replication by targeting the viral nonstructural protein 2. Oncotarget.

[58] Miesen P, Ivens A, Buck AH, van Rij RP (2016). Small RNA profiling in dengue virus 2-infected Aedes Mosquito cells reveals viral piRNAs and novel host miRNAs. PLoS Negl Trop Dis.

[59] Duru N, Zhang Y, Gernapudi R, Wolfson B, Lo PK, Yao Y, Zhou Q (2016). Loss of miR-140 is a key risk factor for radiation-induced lung fibrosis through reprogramming fibroblasts and macrophages. Sci Rep.

[60] Dong W, Yao C, Teng X, Chai J, Yang X, Li B (2016). MiR-140-3p suppressed cell growth and invasion by downregulating the expression of ATP8A1 in non-small cell lung cancer. Tumour Biol.

[61] Song L, Liu H, Gao S, Jiang W, Huang W (2010). Cellular microRNAs inhibit replication of the H1N1 influenza a virus in infected cells. J Virol.

[62] Wang W, Cheng M, Qiao S, Wang Y, Li H, Wang N (2017). Gga-miR-21 inhibits chicken pre-adipocyte proliferation in part by down-regulating Kruppel-like factor 5. Poult Sci.

[63] Wang YS, Ouyang W, Pan QX, Wang XL, Xia XX, Bi ZW, Wang YQ, Wang XM (2013). Overexpression of microRNA gga-miR-21 in chicken fibroblasts suppresses replication of infectious bursal disease virus through inhibiting VP1 translation. Antivir Res.

[64] Wang Y, Brahmakshatriya V, Zhu H, Lupiani B, Reddy SM, Yoon BJ, Gunaratne PH, Kim JH, Chen R, Wang J (2009). Identification of differentially expressed miRNAs in chicken lung and trachea with avian influenza virus infection by a deep sequencing approach. BMC Genomics.

[65] Peng X, Gao QS, Zhou L, Chen ZH, Lu S, Huang HJ, Zhan CY, Xiang M (2015). MicroRNAs in avian influenza virus H9N2-infected and non-infected chicken embryo fibroblasts. Genet Mol Res.

[66] Kemper C, Atkinson JP (2007). T-cell regulation: with complements from innate immunity. Nat Rev Immunol.

[67] Banat GR, Tkalcic S, Dzielawa JA, Jackwood MW, Saggese MD, Yates L, Kopulos R, Briles WE, Collisson EW (2013). Association of the chicken MHC B haplotypes with resistance to avian coronavirus. Dev Comp Immunol.

[68] Bacon LD, Hunter DB, Zhang HM, Brand K, Etches R (2004). Retrospective evidence that the MHC (B haplotype) of chickens influences genetic resistance to attenuated infectious bronchitis vaccine strains in chickens. Avian pathol.

[69] Izumi G, Koga K, Takamura M, Makabe T, Nagai M, Urata Y, Harada M, Hirata T, Hirota Y, Fujii T (2017). Mannose receptor is highly expressed by peritoneal dendritic cells in endometriosis. Fertil Steril.

[70] Lo YL, Liou GG, Lyu JH, Hsiao M, Hsu TL, Wong CH (2016). Dengue virus infection is through a cooperative interaction between a mannose receptor and CLEC5A on macrophage as a multivalent hetero-complex. PLoS One.

[71] Chang WC, White MR, Moyo P, McClear S, Thiel S, Hartshorn KL, Takahashi K (2010). Lack of the pattern recognition molecule mannose-binding lectin increases susceptibility to influenza a virus infection. BMC Immunol.

[72] Michelow IC, Lear C, Scully C, Prugar LI, Longley CB, Yantosca LM, Ji X, Karpel M, Brudner M, Takahashi K (2011). High-dose mannose-binding lectin therapy for Ebola virus infection. J Infect Dis.

[73] Brown KS, Keogh MJ, Owsianka AM, Adair R, Patel AH, Arnold JN, Ball JK, Sim RB, Tarr AW, Hickling TP (2010). Specific interaction of hepatitis C virus glycoproteins with mannan binding lectin inhibits virus entry. Protein Cell.

[74] Tawana K, Rio-Machin A, Preudhomme C, Fitzgibbon J (2017). Familial CEBPA-mutated acute myeloid leukemia. Semin Hematol.

[75] Yu WQ, Sun JN, Tan YH, Cui JW, Li W (2015). recent advances of research on CEBPA mutation in acute myeloid leukemia. Zhongguo shi yan xue ye xue za zhi.

